# Integrated bioinformatic changes and analysis of retina with time in diabetic rats

**DOI:** 10.7717/peerj.4762

**Published:** 2018-05-16

**Authors:** Zekai Cui, Qiaolang Zeng, Yonglong Guo, Shiwei Liu, Jiansu Chen

**Affiliations:** 1 Key Laboratory for Regenerative Medicine, Ministry of Education, Jinan University, Guangzhou, China; 2 The Department of Ophthalmology, The First Clinical Medical College, Jinan University, Guangzhou, China; 3 Institute of Ophthalmology, Medical College, Jinan University, Guangzhou, China; 4 Aier Eye Institute, Changsha, China

**Keywords:** Differentially expressed genes, Gene ontology terms, Protein–protein interaction, Diabetic retinopathy, Bioinformatics

## Abstract

Diabetic retinopathy (DR) is the most common chronic complication of diabetes. It can cause impaired vision and even blindness. However, the pathological mechanism of DR is still unknown. In the present study, we use bioinformatic analysis to reveal the pathological changes of early DR in a streptozotocin (STZ) induced diabetes rat model. The dataset GSE28831 was downloaded from the Gene Expression Omnibus (GEO) database. To clarify the pathological mechanism of early DR, genes which were up-regulated (UP group) or down-regulated (DOWN group) over time were identified. One hundred eighty six genes in the UP group and 85 genes in the DOWN group were defined. There were in total 28 Gene ontology (GO) terms with a *P* value lower than 0.05 in UP group, including astrocyte development, neutrophil chemotaxis, neutrophil aggregation, mesenchymal cell proliferation and so on. In the DOWN group, there were totally 14 GO terms with a *P* value lower than 0.05, including visual perception, lens development in camera-type eye, camera-type eye development, bicellular tight junction and so on. Signaling pathways were analyzed with all genes in the UP and DOWN groups, and leukocyte transendothelial migration and tight junction were selected. Protein–protein interaction (PPI) network was constructed and six hub genes *Diras3*, *Actn1*, *Tssk6*, *Cnot6l*, *Tek* and *Fgf4* were selected with connection degree ≥5. *S100a8*, *S100a9* and *Tek* may be potential targets for DR diagnosis and treatment. This study provides the basis for the diagnosis and treatment of DR in the future.

## Introduction

The number of diabetic patients in the world reached 366 million in 2011. There were 92.4 million diabetic patients in China, ranking first in the world (Yang et al., 2010). The main hazard of diabetes is chronic complications of diabetes. Diabetic retinopathy (DR) is the most common chronic complication of diabetes and is the first blinding eye disease in the working population (Kobrin Klein, 2007). In the American diabetic population, the prevalence of DR was 98% and 78%, respectively, in patients with type 1 and type 2 (Kempen et al., 2004). In the Chinese diabetic population, the prevalence of DR was 37%. Ten to 19 years later, the prevalence of DR increased to 54% (Xie et al., 2009). The Wisconsin Epidemiology Survey of Diabetic Retinopathy (WESDR) found that the blindness rates in patients with type 1 and type 2 diabetes were 3.6% and 1.6%, respectively (Fong et al., 2004). The main causes of visual impairment of DR are diabetic macular edema (DME) and proliferative diabetic retinopathy (PDR), the incidences of which are 23% and 14%, respectively in type 1 and type 2 diabetic patients (Kempen et al., 2004; Porta, Maldari & Mazzaglia, 2011).

The basic pathological process of DR is microcirculatory disturbance. Long-term hyperglycemia leads to vascular endothelial injury, activation of cell adhesion molecules, leukocyte accumulation and activation of a series of cytokines. These changes are followed by the expression of hypoxia regulated growth factors and an increase in cytokines resulting in microcirculatory disturbances, such as intraretinal microvascular abnormalities(IRMA), leakage, obstruction, microaneurysms (Chibber et al., 2007; Kern, 2007). Multiple factor interactions play a key role in the development and progression of DR (Brownlee, 2005).

Streptozotocin (STZ) is particularly toxic to mammalian pancreatic beta cells. Due to its high toxicity to beta cells, streptozotocin has been used in scientific studies to induce insulitis and diabetes in experimental animals (Rossini et al., 1977). Kirwin et al. (2011) used microarrays to evaluate early changes (up to 3 months) in STZ-induced diabetic rats. They found that the expression of visual cyclin proteins was significantly down-regulated post STZ treatment. This microarray dataset (GSE28831) has been uploaded to the Gene Expression Omnibus database. Using this dataset, Zhao et al. (2017) further analyzed differentially expressed genes (DEGs), Gene Ontology (GO) enrichment and signaling pathways on days 7, 28 and 84 in STZ-induced diabetic rats. However, they only analyzed DEGs at this three time points without analyzing the tendency of gene expression over time. The molecule mechanism and pathological process from day 7 to day 84 was still unclear. As a matter of fact, DR is associated with apoptosis, oxidative stress, inflammation and so on. Therefore, the analysis of the integrated bioinformatic changes with time in DR is important.

In previous studies, GSE28831 has been analyzed by two research groups. Kirwin et al. (2011) produced the pathological model and performed microarray analysis. Zhao et al. (2017) also analyzed the dataset, listed Gene Ontology (GO) enrichment terms and pathway analysis at three time points in detail, and analyzed the protein–protein interaction (PPI) of DEGs. To investigate the pathological changes of early stages of DR over time, further analysis was performed with new bioinformatic approach. In this study, we divide the genes in GSE28831 into two groups depending on the increase in expression (UP group) or the decrease in expression (DOWN group) over time. We then analyzed GO enrichment, signaling pathway and protein–protein interaction (PPI) for both groups by comparing DEGs between the respective groups. From these results, the new molecular mechanism and pathological process of early DR are discovered.

## Materials and Methods

### Affymetrix microarray data of the retina in diabetic rats

The data set GSE28831 provided by Kirwin et al. (2011) was downloaded from Gene Expression Omnibus (GEO) (https://www.ncbi.nlm.nih.gov/geo/query/acc.cgi?acc=GSE28831). The preparation of samples is described in their study. They extracted the retinas of Evans rats on days 7, 28 and 84 after STZ induced diabetes. Microarray experiment was performed. Agilent-014879 Whole Rat Genome Microarray 4 × 44 K G4131F was used as the platform.

### Analysis of DEGs over time

The dataset was analyzed with GEO2R. The expression of STZ group was compared with control group. Then, fold-change (FC) was calculated. DEGs in each time point were screened with |log_2_FC| > 1 and *P* value <0.05. In this study, we divided the genes into two groups. If the FC of a gene on day 84 was greater than the FC on day 28 and the FC on day 28 was also greater than the FC on day 7 [FC(7d) <FC(28d) <FC(84d)] and the *P* value of the three time points is at least one less than 0.05, we defined it as a UP gene. Similarly, if the FC on day 84 was less than the FC on day 28 and the FC on day 28 was less than the FC on day 7 [FC(7d) >FC(28d) >FC(84d)], and the *P* value of the three time points was at least one less than 0.05, we defined it as a DOWN gene.

The heatmaps of UP group and DOWN group were designed according to average FC. The data was transformed with log2.

### Functional enrichment analysis

Genes in UP group and DOWN group were submitted to the Database for Annotation, Visualization and Integrated Discovery (DAVID, version 6.8; https://david.ncifcrf.gov/; Huang, Sherman & Lempicki, 2009). Gene ontology (GO) terms and Kyoto Encyclopedia of Genes and Genomes (KEGG) pathways were screened with *P* value <0.05. We selected DEGs of glucose homeostasis, astrocyte development, neutrophil chemotaxis, eye development and bicellular tight junction from GO enrichment results and plotted heatmaps with fold change.

### Protein–protein interaction (PPI) network

The protein–protein interaction (PPI) analysis is necessary to illustrate the molecular mechanisms. In this study, Search Tool for the Retrieval of Interacting Genes (STRING; http://string-db.org/) database was used to construct PPI network. Genes in the UP group and DOWN group were submitted to the database. Interaction score of 0.4 was defined as the screened threshold. Hub genes were selected with connection degree ≥5.

## Results

### Changes of expression in DEGs

**Figure 1:**
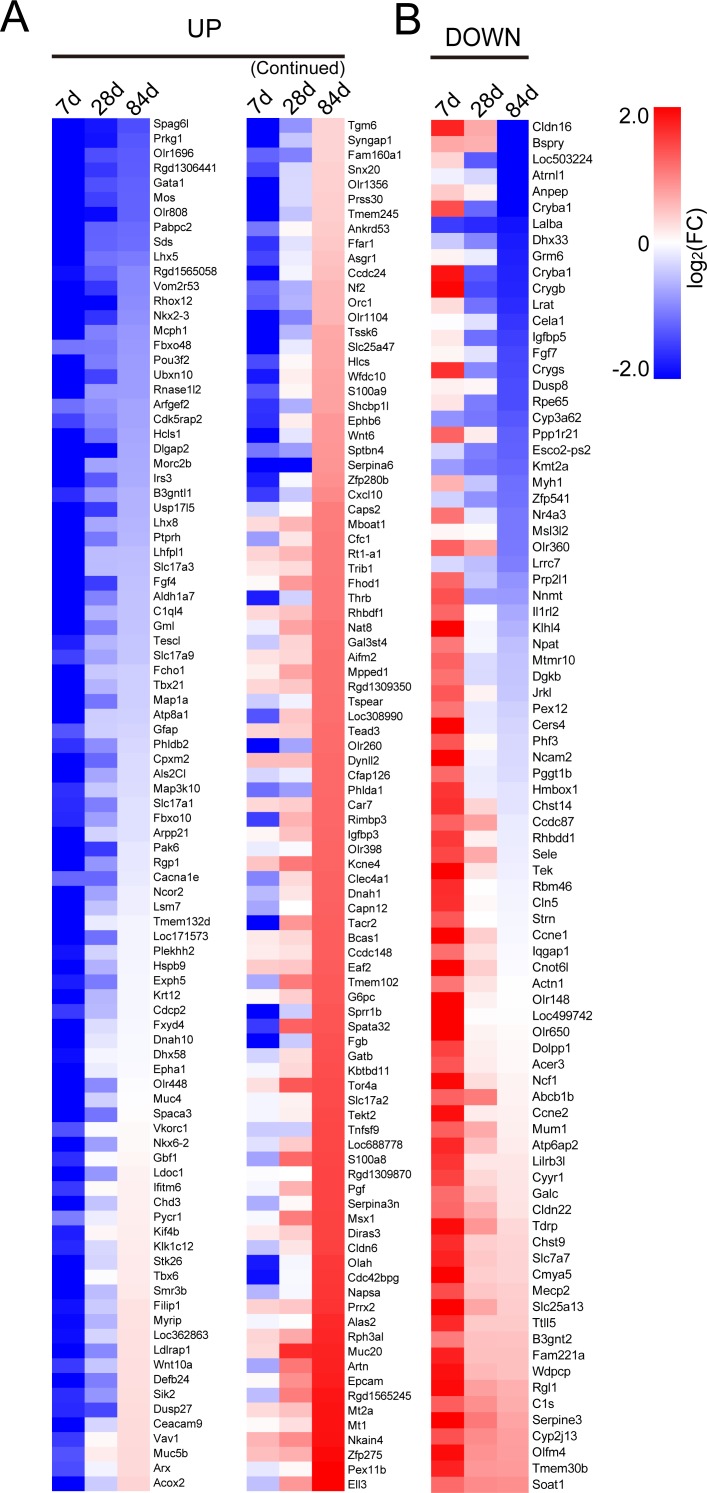
Heatmaps of genes in UP group (A) and DOWN group (B). Red represents up-regulation. Blue represents down-regulation. Each group from left to right are day 7, day 28 and day 84, respectively. Bar unit: log_2_FC.

According to the grouping method mentioned above, 186 genes in UP group and 85 genes in DOWN group were defined. We plotted heatmaps of UP group and DOWN group based on FC of genes (Fig. 1).

### GO terms enrichment and signaling pathways analysis

**Figure 2:**
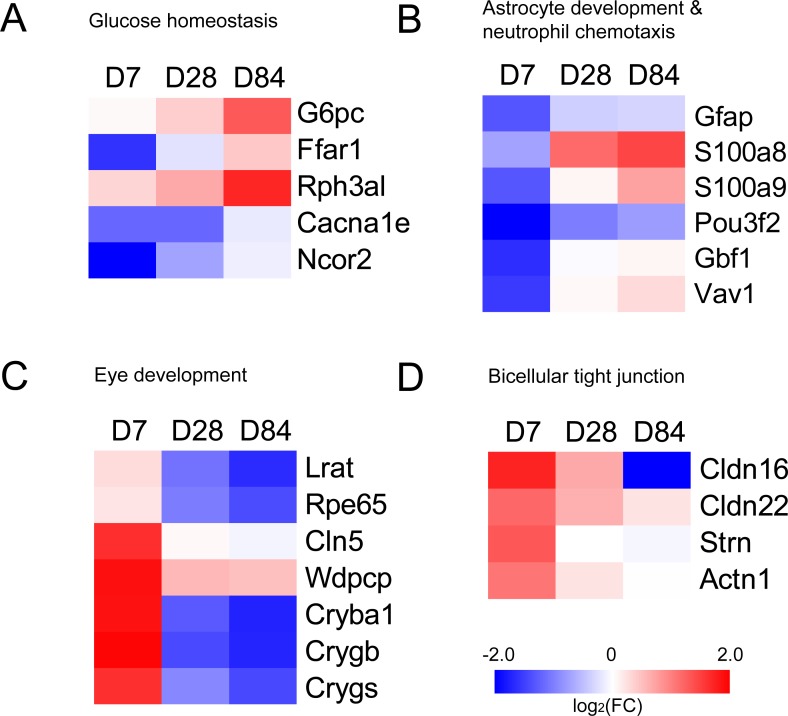
Heatmaps of genes in GO terms (A) glucose homeostasis, (B) astrocyte development & neutrophil chemotaxis, (C) eye development and (D) bicellular tight junction. Red represents up-regulation. Blue represents down-regulation. Each group from left to right are day 7, day 28 and day 84, respectively. Bar unit: log_2_FC.

The genes in UP group and DOWN group were submitted to DAVID to analyze GO term enrichment and KEGG signaling pathway. GO terms consist of Biological Process (BP), Cellular Component (CC) and Molecular Function (MF). There were totally 28 GO terms with a *P* value lower than 0.05 in UP group, including astrocyte development, neutrophil chemotaxis, neutrophil aggregation, mesenchymal cell proliferation and so on (Table 1). In DOWN group, there were totally 14 GO terms with a *P* value lower than 0.05, including visual perception, lens development in camera-type eye, camera-type eye development, bicellular tight junction and so on (Table 2). KEGG signaling pathways were analyzed with all genes in UP group and DOWN group. The screening threshold was set to *P* value <0.05. Only two pathways (leukocyte transendothelial migration and tight junction) were selected (Table 3). We selected DEGs of glucose homeostasis, astrocyte development, neutrophil chemotaxis, eye development and bicellular tight junction from GO enrichment results and plotted heatmaps with fold change (Fig. 2).

**Table 1:** Gene ontology analysis of UP group.

Category	Term	Count	%	*P*-Value	Genes
GO TERM_BP	GO:0042475∼odontogenesis of dentin-containing tooth	6	3.23	0.0003	*Msx1, Nf2, Wnt6, Lhx8, Nkx2-3, Fgf4*
GO TERM_BP	GO:0014002∼astrocyte development	4	2.15	0.0004	*Gfap, S100a8, S100a9, Pou3f2*
GO TERM_BP	GO:0044341∼sodium-dependent phosphate transport	3	1.61	0.0021	*Slc17a3, Slc17a1, Slc17a2*
GO TERM_BP	GO:0046415∼urate metabolic process	3	1.61	0.0027	*G6pc, Slc17a3, Slc17a1*
GO TERM_BP	GO:0021879∼forebrain neuron differentiation	3	1.61	0.0057	*Lhx5, Lhx8, Phlda1*
GO TERM_BP	GO:0021846∼cell proliferation in forebrain	3	1.61	0.0086	*Arx, Lhx5, Ncor2*
GO TERM_BP	GO:0014912∼negative regulation of smooth muscle cell migration	3	1.61	0.0097	*Prkg1, Igfbp3, Trib1*
GO TERM_BP	GO:0035435∼phosphate ion transmembrane transport	3	1.61	0.0108	*Slc17a3, Slc17a1, Slc17a2*
GO TERM_BP	GO:0030593∼neutrophil chemotaxis	4	2.15	0.0147	*Gbf1, S100a8, S100a9, Vav1*
GO TERM_BP	GO:0023019∼signal transduction involved in regulation of gene expression	3	1.61	0.0174	*Epcam, Tbx6, Msx1*
GO TERM_BP	GO:0070488∼neutrophil aggregation	2	1.08	0.0176	*S100a8, S100a9*
GO TERM_BP	GO:0030317∼sperm motility	4	2.15	0.0207	*Spag6l, Tekt2, Dnah1, Cacna1e*
GO TERM_BP	GO:0042593∼glucose homeostasis	5	2.69	0.0237	*G6pc, Ffar1, Rph3al, Cacna1e, Ncor2*
GO TERM_BP	GO:0010273∼detoxification of copper ion	2	1.08	0.0349	*Mt2a, Mt1*
GO TERM_BP	GO:0048863∼stem cell differentiation	3	1.61	0.0362	*Epcam, Msx1, Ell3*
GO TERM_BP	GO:0002793∼positive regulation of peptide secretion	2	1.08	0.0434	*S100a8, S100a9*
GO TERM_BP	GO:0035106∼operant conditioning	2	1.08	0.0434	*Tacr2, Aldh1a7*
GO TERM_BP	GO:0010463∼mesenchymal cell proliferation	2	1.08	0.0434	*Msx1, Fgf4*
GO TERM_BP	GO:0050714∼positive regulation of protein secretion	3	1.61	0.0487	*Fgb, Rph3al, Exph5*
GO TERM_CC	GO:0005615∼extracellular space	19	10.22	0.0318	*Wnt10a, Spaca3, Aifm2, S100a8, Pgf, S100a9, Cpxm2, Artn, Napsa, Tnfsf9, Muc4, Cxcl10, Serpina3n, Serpina6, Fgb, C1ql4, Wnt6, Igfbp3, Muc5b*
GO TERM_MF	GO:0005436∼sodium: phosphate symporter activity	3	1.61	0.0016	*Slc17a3, Slc17a1, Slc17a2*
GO TERM_MF	GO:0015321∼sodium-dependent phosphate transmembrane transporter activity	3	1.61	0.0021	*Slc17a3, Slc17a1, Slc17a2*
GO TERM_MF	GO:0001085∼RNA polymerase II transcription factor binding	4	2.15	0.0137	*Gata1, Tbx6, Hcls1, Tead3*
GO TERM_MF	GO:0003779∼actin binding	7	3.76	0.0226	*Spata32, Myrip, Nf2, Plekhh2, Map1a, Hcls1, Sptbn4*
GO TERM_MF	GO:0017137∼Rab GTPase binding	5	2.69	0.0250	*Myrip, Rph3al, Exph5, Rgp1, Als2cl*
GO TERM_MF	GO:0043565∼sequence-specific DNA binding	11	5.91	0.0281	*Arx, Gata1, Msx1, Thrb, Nkx6-2, Lhx5, Pou3f2, Prrx2, Lhx8, Ncor2, Nkx2-3*
GO TERM_MF	GO:0035662∼Toll-like receptor 4 binding	2	1.08	0.0346	*S100a8, S100a9*
GO TERM_MF	GO:0004672∼protein kinase activity	7	3.76	0.0359	*Stk26, Mos, Map3k10, Prkg1, Sik2, Epha1, Trib1*

**Table 2:** Gene ontology analysis of DOWN group.

Category	Term	Count	%	*P*-value	Genes
GO TERM_BP	GO:0007601∼visual perception	4	4.76	0.0098	*Lrat, Rpe65, Cryba1, Cln5*
GO TERM_BP	GO:0002088∼lens development in camera-type eye	3	3.57	0.0103	*Crygb, Crygs, Cryba1*
GO TERM_BP	GO:0070192∼chromosome organization involved in meiotic cell cycle	2	2.38	0.0311	*Ccne2, Ccne1*
GO TERM_BP	GO:0016051∼carbohydrate biosynthetic process	2	2.38	0.0349	*Chst9, Chst14*
GO TERM_BP	GO:0043010∼camera-type eye development	3	3.57	0.0378	*Wdpcp, Rpe65, Cryba1*
GO TERM_BP	GO:0055123∼digestive system development	2	2.38	0.0387	*Wdpcp, Cela1*
GO TERM_BP	GO:1903827∼regulation of cellular protein localization	2	2.38	0.0425	*Ccne2, Ccne1*
GO TERM_BP	GO:0031100∼organ regeneration	3	3.57	0.0499	*Ccne1, Nr4a3, Nnmt*
GO TERM_CC	GO:0005923∼bicellular tight junction	4	4.76	0.0096	*Cldn16, Strn, Cldn22, Actn1*
GO TERM_CC	GO:0031252∼cell leading edge	3	3.57	0.0215	*Bspry, Actn1, Iqgap1*
GO TERM_CC	GO:0030176∼integral component of endoplasmic reticulum membrane	3	3.57	0.0449	*Acer3, Dolpp1, Rhbdd1*
GO TERM_MF	GO:0005212∼structural constituent of eye lens	3	3.57	0.0034	*Crygb, Crygs, Cryba1*
GO TERM_MF	GO:0001537∼N-acetylgalactosamine 4-O-sulfotransferase activity	2	2.38	0.0197	*Chst9, Chst14*
GO TERM_MF	GO:0045322∼unmethylated CpG binding	2	2.38	0.0313	*Kmt2a, Mecp2*

**Table 3:** KEGG pathway analysis of UP group and DOWN group.

Category	Term	Count	%	*P*-Value	Genes
KEGG_PATHWAY	rno04670: Leukocyte transendothelial migration	6	2.22	0.0228	*Cldn16, Ncf1, Cldn6, Cldn22, Actn1, Vav1*
KEGG_PATHWAY	rno04530: Tight junction	6	2.22	0.0427	*Cldn16, Myh1, Cldn6, Hcls1, Cldn22, Actn1*

**Figure 3:**
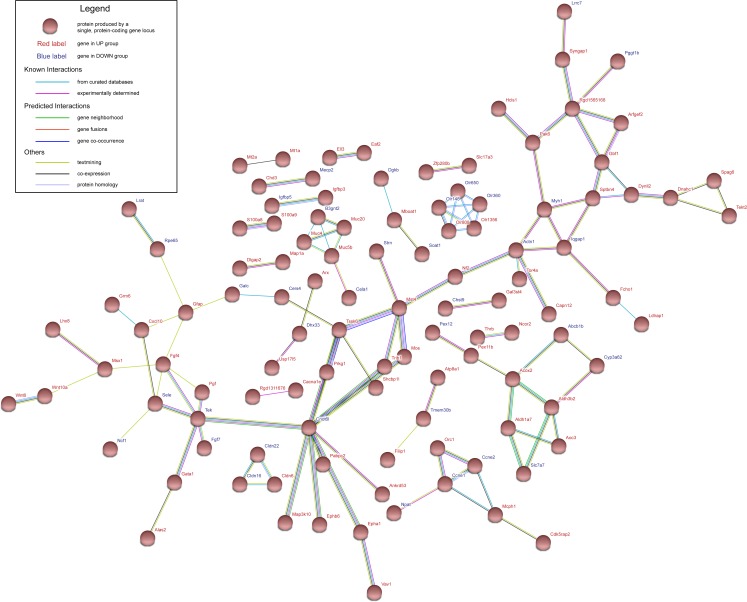
Protein–protein interaction network of UP group and DOWN group. Nodes represent proteins expressed by genes in UP group (red labels) and DOWN group (blue labels).

### Protein–protein interaction (PPI) network construction and hub gene selection

The genes in UP group and DOWN group were submitted to STRING database to construct PPI network. Interaction score of 0.4 was defined as the screened threshold. The constructed PPI network is shown in Fig. 3. Hub genes were selected with connection degree ≥5. Six hub genes were *Diras3*, *Actn1*, *Tssk6*, *Cnot6l*, *Tek and Fgf4 .*

## Discussion

Diabetic retinopathy (DR) is the leading cause of blindness and a major microvasculature complication of diabetes mellitus (DM). At present, the pathogenesis of DR has not been fully elucidated. However, some mechanisms are associated with the pathogenesis of DR.

DM patients have a higher concentration of blood glucose. Hyperglycemia increases the thickness of capillary basement membrane in the nerve fiber layer and the outer plexiform layer, which leads to retinal capillary cell apoptosis, reduced activity of retinal dismutase and catalase (Zhang et al., 2015). Recent studies have shown that hyperglycemia-induced retinal pigment epithelial (RPE) cell apoptosis is also thought to be involved in the development of DR (Kim et al., 2014).

In previous studies, Kirwin et al. (2011) analyzed the microarray data, listed the GO enrichment terms, and discussed the DEGs of the visual cycle in DR. Zhao et al. (2017) analyzed the dataset, listed GO enrichment terms, pathway analysis and PPI at three time points in detail. The effects of genes such as *CYP2B2*, *MASP2*, *LRAT*, *RPE65*, *RDH5*, *MAPK13*, *LRAT* and *RPE65* on DR were discussed. To investigate the pathological changes of early stages of DR over time, further analysis was performed with new bioinformatic approach. In this study, we divided these genes into the UP group and DOWN group based on changes in gene expression. GO terms astrocyte development, neutrophil chemotaxis, neutrophil aggregation, mesenchymal cell proliferation, glucose homeostasis and so on are in the UP group. Visual perception, lens development in camera-type eye, camera-type eye development, bicellular tight junction and so on are in the DOWN group. Meanwhile, signaling pathways such as leukocyte transendothelial migration and tight junction have significant changes. From the above results we can conclude that the following factors play an important role in the development of early DR.

The first one is the abnormality of retinal pigment epithelial (RPE) cells. RPE is the outermost layer of the retina 10-layer structure, located between the choroid and retina. It is composed of a single layer of pigment epithelial cells arranged in a very regular manner. The RPE cells are polygonal. The RPE has many functions, including light absorption, epithelial transport, spatial ion buffering, visual cycle, phagocytosis, secretion and immune modulation (Strauss, 2005). In this study, the expression of *Cldn16* and *Cldn22* was down-regulated over time. Downregulation of claudin, indicated that tight junctions in the retina were damaged. Epithelial-mesenchymal transition (EMT) was occurred. RPE cells undergo EMT and produce fibroblast-like cells, and extracellular matrix (ECM) components, participating in fibrotic sequelae on the detached retina (Saika et al., 2008). In addition, the expression of *Myh1* and *Wdpcp* was down-regulated over time. Myosin II (*Myh1*) is closely related to cell polarity (Vicente-Manzanares et al., 2007). WD repeat containing planar cell polarity effector (*Wdpcp*) can directly modulate the actin cytoskeleton to regulate cell polarity (Cui et al., 2013). RPE cells lose their polarity and remain in the shape of mesenchymal cells, showing deviation from the properties of epithelial cells (Lee et al., 2001). *Ccne1 and Ccne2* were both expressed highly on day 7 and normally on day 84. Cyclin E is a member of the cyclin family. It can regulate EMT through phosphorylation of Slug, a transcriptional repressor (Wang et al., 2015).

The second is the impairment of visual function. The visual cycle is the sensory transduction of the visual system. This is a process by which light transforms into electrical signals in the rod cells, cone cells and photosensitive ganglion cells of the retina. Rpe65 and Lrat are the key enzymes of visual cycle. In this study, their expression was down-regulated over time. Kirwin et al. (2011) demonstrated that in hyperglycemic state, the expression of visual cycle enzymes is down-regulated.

The third is the inflammation in retina. In the present study, the expression of *S100a8* textitand *S100a9* was gradually increased. Calprotectin (*S100A8/A9*), a heterodimer of the two calcium-binding proteins S100A8 and S100A9, was originally discovered as an immunogenic protein expressed and secreted by neutrophils. Subsequently, it has emerged as an important pro-inflammatory mediator in acute and chronic inflammation (Gebhardt et al., 2006). Ryckman et al. (2003) have shown that calprotectin is involved in neutrophil migration to inflammatory sites. Ansari & Ganaie (2014) found that MRP-14 (*S100A9*) protein was upregulated in the ocular microenvironment in patients with PDR, which indicated that increased MRP-14 levels were associated with inflammation in PDR. It is a potential inflammatory marker in PDR. The expression of *Vav1* and *Gbf1* were down-regulated on day 7 and returned to normal or even up-regulated on day 84. They promote neutrophil chemotaxis with calprotectin (Mazaki, Nishimura & Sabe, 2012; Phillipson et al., 2009). Gfap was expressed decreasingly on day 7 and returned to normal on day 84. Gfap is not only involved in retinal damage repair (Humphrey et al., 1997), but is also proposed to play a role in astrocyte-neuron interactions as well as cell–cell communication (Weinstein, Shelanski & Liem, 1991).

The last one is early retinal regeneration. When the retina is damaged, tissue repair and regeneration begin. *Cryba1*, *Crygb* and *Crygs* were highly expressed on day 7, and then decreased on day 28 and 84. βB2-crystallin was shown to be highly expressed in regenerating ganglion cells where they have an autocrine effect promoting retinal ganglion cell axon regrowth (Liedtke et al., 2007). This was among the first evidence suggesting that β-crystallin proteins could also be secreted and be neuroprotective through other mechanisms. A subsequent study then demonstrated that this effect was related to increased inflammation and activation of the CNTF-STAT3 pathway. The authors also showed that γ-crystallins had a similar action (Fischer et al., 2008). Lu et al. (2013) found that *CRYBB2* was significantly elevated in the retina after 1 month of diabetes in mice. A study also revealed that β-crystallin and γ-crystallin were up-regulated in rat’s retina with DR (Fort et al., 2009). Free fatty acid receptor 1 (FFAR1) is an important nutrient sensor of circulating lipids that controls retinal glucose entry to match mitochondrial metabolism with available fuel substrates. Activation of the Ffar1 impairs glucose entry into photoreceptors (Joyal et al., 2016).

PPI network shows six hub genes Diras3, Actn1, Tssk6, Cnot6l, Tek and Fgf4. Diras3 is an imprinted gene, with monoallelic expression of the paternal allele, which is associated with growth suppression. Thus, this gene appears to be a putative tumor suppressor gene whose function is abrogated in ovarian and breast cancers (Yu et al., 2006). A study found that Diras3 inhibits proliferation and activation of NF-κB in glioblastoma (Rymaszewski et al., 2016).Therefore, this gene may become a target of DR treatment and diagnosis. *Actn1* (*α*-Actinin 1) was found to play roles in the survival, motility, and RhoA signaling of astrocytoma cells (Quick & Skalli, 2010). *Cnot6l* was expressed increasingly on day 7 and returned to normal on day 84. Mittal et al. (2011) that Ccr4a (*Cnot6*) and Ccr4b (*Cnot6l*) deadenylase subunits of the human Ccr4-Not complex contribute to the prevention of cell death and senescence. It shows that the death of retinal cells modulated the expression of *Cnot6l* up-regulated. Angiopoietin-1 receptor (*Tek*) is an angiopoietin receptor. In the present study, the expression of *Tek* was up-regulated on day 7 and returned to normal on day 84. Khalaf & Helmy (2017) found that the angiopoietin/tie system and VEGF are essential features in the commencement and development of PDR. AKB-9778 is a small-molecule that promotes Tie2 activation. In clinical trials, patients with diabetic macular edema (DME) were treated with AKB-9778 for 4 weeks. It reduced macular edema and improved vision in some patients (Campochiaro et al., 2015). In 2016, this research group found that in the clinical trial the combination of Tie2 activator AKB-9778 and vascular endothelial growth factor (VEGF) inhibitor ranibizumab was significantly more effective than suppression of VEGF alone in reducing DME (Campochiaro et al., 2016). So Tie2 (*Tek*) may be an important factor of DR development.

This rat model is STZ-induced. No literature was found to prove that STZ can directly affect the expression of the above genes. However, the toxicity of STZ can cause DNA damage, chromosome aberrations, and cell death (Bolzán & Bianchi, 2002). STZ also has more or less illimitable effects on nervous system (Biessels et al., 1999), cardiovascular system (Schaan et al., 2004), kidneys (Martin et al., 2004), respiratory system (Samarghandian, Afshari & Sadati, 2014), and reproductive system (Ansari & Ganaie, 2014). These effects may lead to indirect changes in gene expression. It needs further proof of study.

## Conclusions

In conclusion, we speculate that the following pathological mechanisms of STZ-induced diabetes rat model with the above microarray analysis and prediction of key genes. On day 7, the retina was firstly damaged and RPE EMT occurred. Then crystallin repaired early damaged ganglion cells. On day 84, the expression of key enzyme of visual cycle was down-regulated. Inflammation occurred and EMT gradually stopped. Neutrophil chemotaxis occurred. Polarity and function of epithelial and endothelial cell were lost. In addition, *S100a8*, *S100a9* and *Tek* may be potential targets for DR diagnosis and treatment. This provides the basis for the diagnosis and treatment of DR in the future.

##  Supplemental Information

10.7717/peerj.4762/supp-1 Supplemental Information 1 The information of each sample

10.7717/peerj.4762/supp-2 Supplemental Information 2 The expression of each gene in samples

## Additional Information and Declarations

### Microarray Data Deposition

The following information was supplied regarding the deposition of microarray data:
GSE28831

### Data Availability

The following information was supplied regarding data availability: The raw data are provided in the Supplemental Files.

## References

[ref-1] Abu El-Asrar AM, Alam K, Siddiquei MM, Van den Eynde K, Mohammad G, Hertogh GD, Opdenakker G (2016). Myeloid-Related Protein-14/MRP-14/S100A9/Calgranulin B is associated with inflammation in proliferative diabetic retinopathy. Ocular Immunology and Inflammation.

[ref-2] Ansari MN, Ganaie MA (2014). Ameliorative effect of rocket leaves on fertility in streptozotocin-induced diabetic rats. International Research Journal of Biological Sciences.

[ref-3] Biessels G-J, Cristino NA, Rutten G-J, Hamers FP, Erkelens DW, Gispen WH (1999). Neurophysiological changes in the central and peripheral nervous system of streptozotocin-diabetic rats: course of development and effects of insulin treatment. Brain.

[ref-4] Bolzán AD, Bianchi MS (2002). Genotoxicity of streptozotocin. Mutation Research/Reviews in Mutation Research.

[ref-5] Brownlee M (2005). The pathobiology of diabetic complications. Diabetes.

[ref-6] Campochiaro PA, Khanani A, Singer M, Patel S, Boyer D, Dugel P, Kherani S, Withers B, Gambino L, Peters K (2016). Enhanced benefit in diabetic macular edema from AKB-9778 Tie2 activation combined with vascular endothelial growth factor suppression. Ophthalmology.

[ref-7] Campochiaro PA, Sophie R, Tolentino M, Miller DM, Browning D, Boyer DS, Heier JS, Gambino L, Withers B, Brigell M (2015). Treatment of diabetic macular edema with an inhibitor of vascular endothelial-protein tyrosine phosphatase that activates Tie2. Ophthalmology.

[ref-8] Chibber R, Ben-Mahmud BM, Chibber S, Kohner EM (2007). Leukocytes in diabetic retinopathy. Current Diabetes Reviews.

[ref-9] Cui C, Chatterjee B, Lozito TP, Zhang Z, Francis RJ, Yagi H, Swanhart LM, Sanker S, Francis D, Yu Q (2013). Wdpcp, a PCP protein required for ciliogenesis, regulates directional cell migration and cell polarity by direct modulation of the actin cytoskeleton. PLOS Biology.

[ref-10] Fischer D, Hauk TG, Müller A, Thanos S (2008). Crystallins of the β/γ-superfamily mimic the effects of lens injury and promote axon regeneration. Molecular and Cellular Neuroscience.

[ref-11] Fong DS, Aiello L, Gardner TW, King GL, Blankenship G, Cavallerano JD, Ferris FL, Klein R (2004). Retinopathy in diabetes. Diabetes Care.

[ref-12] Fort PE, Freeman WM, Losiewicz MK, Singh RS, Gardner TW (2009). The retinal proteome in experimental diabetic retinopathy up-regulation of crystallins and reversal by systemic and periocular insulin. Molecular & Cellular Proteomics.

[ref-13] Gebhardt C, Németh J, Angel P, Hess J (2006). S100A8 and S100A9 in inflammation and cancer. Biochemical Pharmacology.

[ref-14] Huang DW, Sherman BT, Lempicki RA (2009). Systematic and integrative analysis of large gene lists using DAVID bioinformatics resources. Nature Protocols.

[ref-15] Humphrey MF, Chu Y, Mann K, Rakoczy P (1997). Retinal GFAP and bFGF expression after multiple argon laser photocoagulation injuries assessed by both immunoreactivity and mRNA levels. Experimental Eye Research.

[ref-16] Joyal J, Sun Y, Gantner ML, Shao Z, Evans LP, Saba N, Burnim S, Jin SK, Patel G (2016). Retinal lipid and glucose metabolism dictates angiogenesis through lipid sensor Ffar1. Nature Medicine.

[ref-17] Kempen JH, O’colmain B, Leske MC, Haffner S, Klein R, Moss S, Taylor H, Hamman R (2004). The prevalence of diabetic retinopathy among adults in the United States. Archives of Ophthalmology.

[ref-18] Kern TS (2007). Contributions of inflammatory processes to the development of the early stages of diabetic retinopathy. Journal of Diabetes Research.

[ref-19] Khalaf N, Helmy H (2017). Role of angiopoietins and Tie-2 in diabetic retinopathy. Electronic Physician.

[ref-20] Kim D-I, Park M-J, Lim S-K, Choi J-H, Kim J-C, Han H-J, Kundu TK, Park J-I, Yoon K-C, Park S-W (2014). High-glucose-induced CARM1 expression regulates apoptosis of human retinal pigment epithelial cells via histone 3 arginine 17 dimethylation: role in diabetic retinopathy. Archives of Biochemistry and Biophysics.

[ref-21] Kirwin SJ, Kanaly ST, Hansen CR, Cairns BJ, Ren M, Edelman JL (2011). Retinal gene expression and visually evoked behavior in diabetic long evans rats. Investigative Ophthalmology & Visual Science.

[ref-22] Kobrin Klein BE (2007). Overview of epidemiologic studies of diabetic retinopathy. Ophthalmic Epidemiology.

[ref-23] Lee S-C, Kwon O-W, Seong G-J, Kim S-H, Ahn J-E, Kay E-DP (2001). Epitheliomesenchymal transdifferentiation of cultured RPE cells. Ophthalmic Research.

[ref-24] Liedtke T, Schwamborn JC, Schröer U, Thanos S (2007). Elongation of axons during regeneration involves retinal crystallin β b2 (crybb2). Molecular & Cellular Proteomics.

[ref-25] Lu L, Seidel CP, Iwase T, Stevens RK, Gong YY, Wang X, Hackett SF, Campochiaro PA (2013). Suppression of GLUT1; A new strategy to prevent diabetic complications. Journal of Cellular Physiology.

[ref-26] Martin PM, Roon P, Van Ells TK, Ganapathy V, Smith SB (2004). Death of retinal neurons in streptozotocin-induced diabetic mice. Investigative Ophthalmology & Visual Science.

[ref-27] Mazaki Y, Nishimura Y, Sabe H (2012). GBF1 bears a novel phosphatidylinositol-phosphate binding module, BP3K, to link PI3Kγ activity with Arf1 activation involved in GPCR-mediated neutrophil chemotaxis and superoxide production. Molecular Biology of the Cell.

[ref-28] Mittal S, Aslam A, Doidge R, Medica R, Winkler GS (2011). The Ccr4a (CNOT6) and Ccr4b (CNOT6L) deadenylase subunits of the human Ccr4–Not complex contribute to the prevention of cell death and senescence. Molecular Biology of the Cell.

[ref-29] Phillipson M, Heit B, Parsons SA, Petri B, Mullaly SC, Colarusso P, Gower RM, Neely G, Simon SI, Kubes P (2009). Vav1 is essential for mechanotactic crawling and migration of neutrophils out of the inflamed microvasculature. The Journal of Immunology.

[ref-30] Porta M, Maldari P, Mazzaglia F (2011). New approaches to the treatment of diabetic retinopathy. Diabetes, Obesity and Metabolism.

[ref-31] Quick Q, Skalli O (2010). α-Actinin 1 and α-actinin 4: contrasting roles in the survival, motility, and RhoA signaling of astrocytoma cells. Experimental Cell Research.

[ref-32] Rossini AA, Like AA, Chick WL, Appel MC, Cahill GF (1977). Studies of streptozotocin-induced insulitis and diabetes. Proceedings of the National Academy of Sciences of the United States of America.

[ref-33] Ryckman C, Vandal K, Rouleau P, Talbot M, Tessier PA (2003). Proinflammatory activities of S100: proteins S100A8, S100A9, and S100A8/A9 induce neutrophil chemotaxis and adhesion. The Journal of Immunology.

[ref-34] Rymaszewski A, Straza M, Frei A, Bergom C (2016). The tumor suppressive small GTPase DiRas3 (ARHI) inhibits proliferation and activation of NF-κB in glioblastoma. AACR.

[ref-35] Saika S, Yamanaka O, Flanders KC, Okada Y, Miyamoto T, Sumioka T, Shirai K, Kitano A, Miyazaki K-I, Tanaka S-I (2008). Epithelial-mesenchymal transition as a therapeutic target for prevention of ocular tissue fibrosis. Endocrine, Metabolic & Immune Disorders-Drug Targets (Formerly Current Drug Targets-Immune, Endocrine & Metabolic Disorders).

[ref-36] Samarghandian S, Afshari R, Sadati A (2014). Evaluation of lung and bronchoalveolar lavage fluid oxidative stress indices for assessing the preventing effects of safranal on respiratory distress in diabetic rats. The Scientific World Journal.

[ref-37] Schaan B, Dall’Ago P, Maeda CY, Ferlin E, Fernandes T, Schmid H, Irigoyen M (2004). Relationship between cardiovascular dysfunction and hyperglycemia in streptozotocin-induced diabetes in rats. Brazilian Journal of Medical and Biological Research.

[ref-38] Strauss O (2005). The retinal pigment epithelium in visual function. Physiological Reviews.

[ref-39] Vicente-Manzanares M, Zareno J, Whitmore L, Choi CK, Horwitz AF (2007). Regulation of protrusion, adhesion dynamics, and polarity by myosins IIA and IIB in migrating cells. The Journal of Cell Biology.

[ref-40] Wang W, Huang H, Kao S, Hsu Y, Wang Y, Li K, Chen Y, Yu S, Wang S, Hsiao T (2015). Slug is temporally regulated by cyclin E in cell cycle and controls genome stability. Oncogene.

[ref-41] Weinstein DE, Shelanski ML, Liem R (1991). Suppression by antisense mRNA demonstrates a requirement for the glial fibrillary acidic protein in the formation of stable astrocytic processes in response to neurons. The Journal of Cell Biology.

[ref-42] Xie X, Xu L, Jonas J, Wang Y (2009). Prevalence of diabetic retinopathy among subjects with known diabetes in China: the Beijing Eye Study. European Journal of Ophthalmology.

[ref-43] Yang W, Lu J, Weng J, Jia W, Ji L, Xiao J, Shan Z, Liu J, Tian H, Ji Q (2010). Prevalence of diabetes among men and women in China. New England Journal of Medicine.

[ref-44] Yu Y, Luo R, Lu Z, Feng WW, Badgwell D, Issa JP, Rosen DG, Liu J, Bast RC (2006). Biochemistry and biology of ARHI (DIRAS3), an imprinted tumor suppressor gene whose expression is lost in ovarian and breast cancers. Methods in Enzymology.

[ref-45] Zhang L, Xia H, Han Q, Chen B (2015). Effects of antioxidant gene therapy on the development of diabetic retinopathy and the metabolic memory phenomenon. Graefe’s Archive for Clinical and Experimental Ophthalmology.

[ref-46] Zhao W, Wang D, Zhao J, Zhao W (2017). Bioinformatic analysis of retinal gene function and expression in diabetic rats. Experimental and Therapeutic Medicine.

